# DNA methylation profiling in doxorubicin treated primary locally advanced breast tumours identifies novel genes associated with survival and treatment response

**DOI:** 10.1186/1476-4598-9-68

**Published:** 2010-03-25

**Authors:** Emelyne Dejeux, Jo Anders Rønneberg, Hiroko Solvang, Ida Bukholm, Stephanie Geisler, Turid Aas, Ivo G Gut, Anne-Lise Børresen-Dale, Per Eystein Lønning, Vessela N Kristensen, Jörg Tost

**Affiliations:** 1 Laboratory for Epigenetics, Centre National de Génotypage, CEA - Institut de Génomique, Evry, France; 2 Department of Genetics, Institute for Cancer Research, Norwegian Radium Hospital, Rikshospitalet University Hospital Montebello, Oslo, Norway; 3 Faculty of Medicine, University of Oslo, Norway; 4 Institute for Medical Statistics, University of Oslo, Norway; 5 Department of Surgery, Akerhus University Hospital, Lørenskog, Norway; 6 Department of Oncology, Haukeland University Hospital, Bergen, Norway; 7 Department of Surgery, Haukeland University Hospital, Bergen, Norway; 8 Section of Oncology, Institute of Medicine, University of Bergen, Norway

## Abstract

**Background:**

Breast cancer is the most frequent cancer in women and consists of a heterogeneous collection of diseases with distinct histopathological, genetic and epigenetic characteristics. In this study, we aimed to identify DNA methylation based biomarkers to distinguish patients with locally advanced breast cancer who may benefit from neoadjuvant doxorubicin treatment.

**Results:**

We investigated quantitatively the methylation patterns in the promoter regions of 14 genes (*ABCB1*, *ATM*, *BRCA1*, *CDH3*, *CDKN2A*, *CXCR4*, *ESR1*, *FBXW7*, *FOXC*1, *GSTP1*, *IGF2*, *HMLH1*, *PPP2R2B*, and *PTEN*) in 75 well-described pre-treatment samples from locally advanced breast cancer and correlated the results to the available clinical and molecular parameters. Six normal breast tissues were used as controls and 163 unselected breast cancer cases were used to validate associations with histopathological and clinical parameters.

Aberrant methylation was detected in 9 out of the 14 genes including the discovery of methylation at the *FOXC1 *promoter. Absence of methylation at the *ABCB1 *promoter correlated with progressive disease during doxorubicin treatment. Most importantly, the DNA methylation status at the promoters of *GSTP1*, *FOXC1 *and *ABCB1 *correlated with survival, whereby the combination of methylated genes improved the subdivision with respect to the survival of the patients. In multivariate analysis *GSTP1 *and *FOXC1 *methylation status proved to be independent prognostic markers associated with survival.

**Conclusions:**

Quantitative DNA methylation profiling is a powerful tool to identify molecular changes associated with specific phenotypes. Methylation at the *ABCB1 *or *GSTP1 *promoter improved overall survival probably due to prolonged availability and activity of the drug in the cell while *FOXC1 *methylation might be a protective factor against tumour invasiveness. *FOXC1 *proved to be general prognostic factor, while *ABCB1 *and *GSTP1 *might be predictive factors for the response to and efficacy of doxorubicin treatment. Pharmacoepigenetic effects such as the reported associations in this study provide molecular explanations for differential responses to chemotherapy and it might prove valuable to take the methylation status of selected genes into account for patient management and treatment decisions.

## Background

Breast cancer, the most frequent cancer in women, consists of a heterogeneous collection of diseases with distinct histopathological, genetic and epigenetic characteristics [1]. Conventional single parameters as well as gene expression signatures have been correlated to breast cancer prognosis. However, in contrast to endocrine therapy for which estrogen receptor expression is a predictive marker of response to therapy, we so far lack predictive factors for the selection of a chemotherapeutic regime except for ERBB2 (HER-2) overexpression advocating trastuzumab and increased anthracycline dosing [2].

While the contribution of genetic factors to breast carcinogenesis has long been recognized, it has become evident that epigenetic changes leading to transcriptional silencing of tumour suppressor genes are an at least equally contributing mechanism. In tumours a global loss of DNA methylation (hypomethylation) of the genome is observed at early stages of breast carcinogenesis which proceeds with increasing malignancy [3]. The overall decrease in DNA methylation is accompanied by a gene-specific increase of methylation (hypermethylation) of multiple promoter associated CpG islands leading to transcriptional silencing of genes involved in cell cycle arrest as well as apoptosis [4,5].

The number of genes that has been identified to be aberrantly methylated in breast cancer is rapidly growing. Thus, high-throughput DNA methylation mapping technologies have the potential to identify distinct methylation signatures correlating with specific clinical stages and subtypes, but also to reveal the large heterogeneity of DNA methylation patterns within a tumour subgroup [6-9]. Considering the need to improve prognostication in breast cancer in general, and drug sensitivity prediction in particular [2], the examination of epigenetic gene alterations may improve our knowledge about the outcome and the response of a patient to given treatment.

Recently, we reported the haplotype structure to influence the level of DNA methylation of the *GSTP1 *promoter in breast cancers and to affect patient survival [10]. Here we broaden our analysis studying the methylation patterns in the promoter regions of 14 genes in 75 pre-treatment samples from locally advanced breast cancer by pyrosequencing. Genes were selected on the following basis: 1. previous reports of DNA methylation in breast tumours or at least breast cancer cell lines (*ABCB1 *[11], *ATM *[12], *BRCA1 *[13], *CDH3 *[14], *CDKN2A *[13], *ESR1 *[15], *GSTP1 *[16], *IGF2 *[17], *HMLH1 *[13], *PPP2R2B *[18], *PTEN *[19]) or other cancers (*CXCR4 *[20]), 2. genes displaying variation in breast cancer gene expression profiles (*FOXC1 *[21]) and 3. tumour suppressor genes known to harbour somatic mutations or be situated in frequently deleted regions in breast cancer but for which no DNA methylation analysis has so far been performed (*FBXW7 *[22]). In total 432 CpG positions were investigated resulting in a data set of more than 37.000 quantitative epigenotypes, confirming previously reported associations and identifying novel DNA methylation based biomarkers associated with response to treatment and survival.

## Results

**Figure 1:**
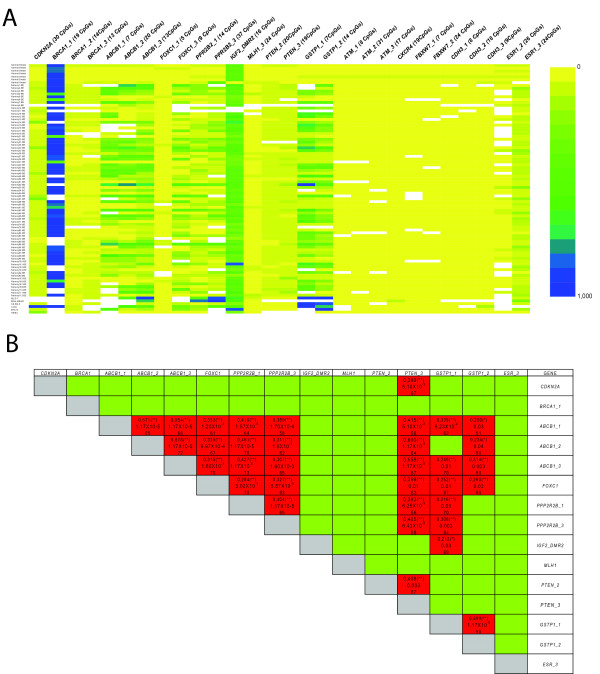
**Summary of the methylation data**. A) Summary of the average DNA methylation values in percentage for the analysis of the fourteen genes (x-axis) in the 75 breast cancer samples, six normal breast tissues (on top) and the six breast cancer cell lines (bottom lines). Absence of methylation over an amplification product is shown in yellow, complete methylation in dark blue; intermediate methylation degrees by the corresponding mixtures of the two colours. B) Nonparametric correlation of methylation levels between genes and between regions within the same gene. The first row and the last column contain the gene name or gene name followed by a number that indicate different genomic regions within the same gene (Additional File 5). Green squares have been assigned to correlations that are non significant. Red square correlations are significant after FDR correction (threshold 10%). For each significant correlation 3 values are given from top to bottom: the correlation coefficient (R^2 ^value), the p-value and the number of tested samples.

We analyzed promoter methylation at 432 CpGs in 14 genes giving rise to 37.000 epigenotypes (Figure 1A). The analysis included the DNA methylation in *ABCB1 *(40 CpGs), *ATM *(56 CpGs), *BRCA1 *(46 CpGs), *CDH3 *(35 CpGs), *CDKN2A *(30 CpGs), *CXCR4 *(19 CpGs), *ESR1 *(50 CpGs) *FBXW7 *(31 CpGs), *FOXC1 *(14 CpGs), *GSTP1 *(21 CpGs), *IGF2 *(16 CpGs), *MLH1 *(24 CpGs), *PPP2R2B *(51 CpGs), and *PTEN *(39 CpGs). The six normal samples were unmethylated for all analyzed regions except for the highly methylated upstream region of *BRCA1*, the differentially methylated region of the imprinted *IGF2 *and the promoter region of *ESR1 *(Figure 1A). Three amplification products in the *ABCB1 *gene were found to be methylated in 70%, 64% and 81% of the tumours. Methylation was found for *CDKN2A *(34% of the samples), *FOXC1 *(50%), *PPP2R2B *(56% and 65%), *HMLH1 *(14%), *PTEN *(22% and 76%) and *GSTP1 *(65% and 83%). All samples were unmethylated for the transcription start site of *BRCA1*, *ATM*, *CDH3*, *CXCR4 *and *FBXW7*. 10% of the samples exhibited a significant hypomethylation in the far upstream region of the *BRCA1 *CpG island. Some methylation was found around the transcription start site for *ESR1 *but also within the normal breast samples. None of the genes displayed an age-dependent variation of DNA methylation at the analyzed loci.

Restricting the analysis to the loci with variable DNA methylation levels, no sample showed a completely normal methylation profile, 3/75 tumours (4%) showed abnormal methylation at one locus, 8 (11%) at two loci, 4 (5%) at three, 14 (19%) at four and five loci, respectively, 16 (21%) at six, 9 (12%) at seven, 5 (7%) at eight loci and two tumours (3%) displayed aberrant methylation at nine loci. On average, five loci were thus aberrantly methylated in any sample. Methylation events at the different loci were not randomly distributed and independent from each other (Figure 1B). As expected, the methylation degrees of the different regions of the same gene were highly correlated if methylation was detected in all amplification products. Less expected, concomitant methylation was often found at different genes such as the *ABCB1*, *FOXC1*, *GSTP1*, *PPP2R2B*, *PTEN *promoters identifying thus strongly correlated methylation events on different chromosomes (Figure 1B). Methylation at the estrogen receptor promoter did not correlate with any other gene. Pyrosequencing provides the advantage of yielding highly quantitative data on consecutive CpGs permitting analysis of the homogeneity of the methylation profiles. We identified for most genes some "core"-regions where DNA methylation levels correlated best with molecular and clinical parameters (see below). For most genes, these regions spanned - as expected - the transcription start sites.

### Correlation to expression profiles

The observed methylation patterns were compared to the tumour subclasses as defined by microarray expression profiling [21]. Basal-like tumours generally showed a lower degree of methylation than the other subclasses (luminal A, luminal B, ERBB2 and normal-like). There was a trend for the absence of methylation at *ABCB1*, *FOXC1*, *PPP2R2B*, and *GSTP1 *in both the basal-like and normal-like tumours, while *IGF2*, *MLH1 *and *PTEN *were hypomethylated in the basal-like tumours but not in the normal-like tumours. When analyzing the correlation between the expression level and the DNA methylation status of individual genes, genes with methylated promoters were almost exclusively not expressed, while unmethylated genes could be expressed as well as not be expressed weakening the correlation. The only significant correlation was obtained for *GSTP1 *(p = 0.003, Correlation coefficient -0.47). Because of their association with survival (see below) we analyzed the expression levels of *GSTP1*, *ABCB1 *and *FOXC1 *by qRT-PCR (Additional File 1). qRT-PCR analysis correlated well with the microarray data (*GSTP1*: Pearson Corr. 0.661, p = 0.003; *FOXC1*: Pearson Corr. 0.788, p < 0.001; *ABCB1*: Pearson Corr. 0.739, p = 0.015). Consequently a significant negative correlation between expression as measured by TaqMan and methylation was found for *GSTP1 *(Spearman Correlation -0.567, p = 0.018), while expression and methylation for *FOXC1 *and *ABCB1 *were not significantly correlated (p = 0.5 and p = 0.368, respectively). Highly expressing genes were unmethylated for the respective promoter region of *GSTP1 *and *FOXC1 *and methylated promoters correlated with silenced expression. The weak correlation between expression and DNA methylation for *FOXC1 *was due to the fact that the gene was already silenced in most tumours independent of its methylation status. Four samples were methylated for *ABCB1 *but displayed high expression. This might be due to alternative usage of an upstream promoter [23] that is not under the control of the analyzed CpG island.

### Correlation with clinical parameters

Methylation was analyzed in the discovery and validation cohorts both as a categorical variable, i.e. the presence/absence of methylation at the respective promoter in association with the tumour characteristics, and as a quantitative variable investigating potential associations between the extent or the distribution of DNA methylation values and the analyzed clinical and molecular parameters (Additional File 2). Promoter methylation of *PPP2R2B *in the pre-treatment sample was significantly associated with tumour grade (p = 0.019), whereby high-grade tumours were more frequently unmethylated than grade 1 and 2 tumours in the discovery cohort. The same was observed in the validation cohort of unselected breast cancers (p = 0.008). No association between methylation and tumour size was found. Estrogen receptor status positivity was associated with the presence and increased extent of methylation at the *PPP2R2B *promoter in both the discovery (p = 0.004) and the validation cohort (p = 0.006). Samples unmethylated for *ABCB1 *and those with increased levels of methylation in the differentially methylated region 2 of *IGF2 *had more often overexpression of the *ERBB2 *oncogene (p = 0.005 and p = 0.007, respectively), previously analyzed by immunohistochemistry [24]. No ERBB2 data was available for the validation cohort.

### Correlation with *TP53 *mutations

We compared the observed DNA methylation profiles with the *TP53 *mutations status and found the lack of *ABCB1 *and *PPP2R2B *methylation to be associated with the presence of *TP53 *mutations in the discovery cohort (p = 0.028 and p = 0.010, respectively) as well as in the validation cohort (p = 0.018 and p = 0.001, respectively). Tumours unmethylated for the middle region of the *ABCB1 *CpG island were associated with mutations in the loop domains L2/L3 (p = 0.022), a region that has previously been shown to be associated with lack of response to doxorubicin based treatment. *PPP2R2B *did not show any differential degree of methylation in function of the type of *TP53 *mutation.

### Survival analysis and response to treatment in the doxorubicin treated cohort

**Figure 2:**
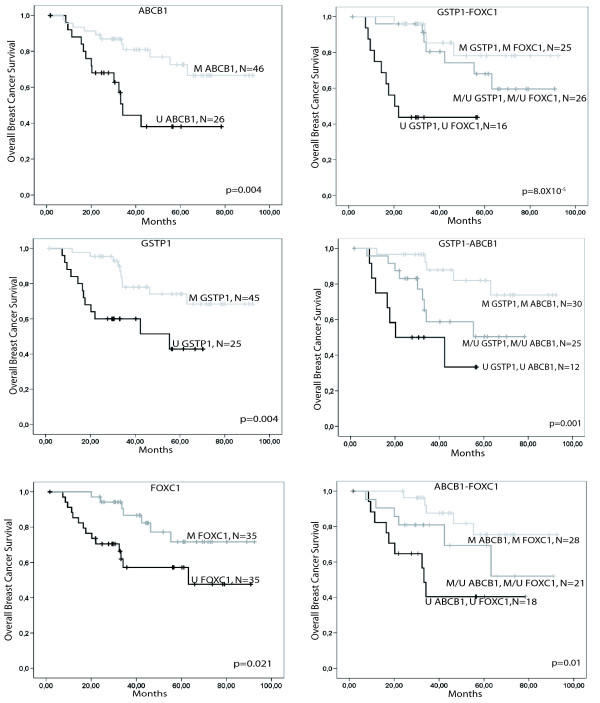
**Kaplan-Meyer plots of overall survival**. Kaplan-Meyer plots of overall survival for patients with either methylated or unmethylated *GSTP1*, *FOXC1 *or *ABCB1 *promoter, respectively (left column). Increased differentiation of patients is obtained through the use of two gene methylation panels, having both genes methylated, either of the two genes methylated and the other one unmethylated or both genes unmethylated (right column). The p-value was calculated using a log rank test and are given uncorrected. 'N' is the number of samples in each group. After Bonferroni correction, DNA methylation of *GSTP1 *and *ABCB1 *as well as *GSTP1/ABCB1 *and *GSTP1/FOXC1 *did reach the level of statistical significance. Deaths due to causes not related to breast cancer were censored.

The eight genes displaying variable DNA methylation patterns in a significant number of tumours (*ABCB1*, *BRCA1*, *CDKN2A*, *FOXC1*, *GSTP1*, *IGF2*, *PPP2R2B *and *PTEN*) within the discovery cohort were tested for association with survival by a logrank test. Breast cancer specific survival was significantly improved in patients with methylated promoters for *ABCB1*, *GSTP1 *and *FOXC1 *(p = 0.004, p = 0.004 and p = 0.021 respectively, Figure 2). Methylation of *ABCB1 *and *GSTP1 *did also reach statistical significance after correction for multiple testing (Bonferroni correction, uncorrected p < 0.00625). Consistently, absence of methylation (p = 0.0076, Additional File 2) in the CpG island of *ABCB1 *was associated with poor response to doxorubicin (progressive disease) in the patient cohort treated with doxorubicin. In the validation cohort treated with different regimens, a significant difference in survival between methylated and unmethylated samples was confirmed for *FOXC1 *(p = 0.024) with patients unmethylated for the promoter region having again worse survival. Methylation of *GSTP1 *did not condition improved survival in the validation cohort of patients (p = 0.331). Similarly, only a trend for improved survival was observed for the methylation status of *ABCB1 *(p = 0.070). The findings for *GSTP1 *and *ABCB1 *might indicate a treatment specific effect on survival

Survival analysis in the doxorubicin treated cohort based on the logrank test indicated that *TP53 *mutation status (p = 0.001), grade (p = 0.001) and the estrogen receptor status (p = 0.002) could slightly better differentiate two survival groups in the analyzed sample collection when compared to the methylation status of the single genes (*ABCB1 *(p = 0.004), *GSTP1 *(p = 0.004) and *FOXC1 *(p = 0.021)), while separation based on the progesterone receptor status and amplification of *ERBB2 *or *TOP2A *did not reach statistical significance. However, combination of two of the discovered DNA methylation markers further improved the distinction between doxorubicin treated patients having two, one or none of the genes methylated. No statistical difference on survival in function of the gene was found when comparing patients that had one of the two genes methylated and these were therefore combined for analysis. The best two-gene methylation pair comprised *GSTP1 *and *FOXC1 *(p = 8·10^-5^), followed by *GSTP1 *and *ABCB1 *(p = 0.001) and *ABCB1 *and *FOXC1 *(p = 0.01). Patients with all three genes methylated (n = 20) had an improved survival compared to patients with all three genes unmethylated (n = 10, p = 0.001). However, the separation lost its statistical significance when patients with mixed methylation patterns for all three genes were added to the analysis. We investigated if expression could be used as an alternative molecular measure to DNA methylation and divided samples in high versus low expression based on the mean expression values. The expression of *GSTP1 *was significantly associated with survival with patients with low levels of expression having as expected an improved survival (p = 0.048). *FOXC1 *(p = 0.247) and *ABCB1 *(p = 0.181) were not significant but again showed improved survival for low expressing patients. When combining DNA methylation and expression, patients with an unmethylated *GSTP1 *promoter and expressed gene had poorer survival compared to patients with a methylated promoter that did not express *GSTP1 *(p = 0.047). The same correlation was observed for *FOXC1 *(p = 0.045) and *ABCB1 *(p = 0.022).

### Cox regression analysis of methylation markers and clinical variables in the doxorubicin treated cohort

To identify significant parameters contributing to the observed differences in survival, Cox regression analysis was performed. The hazard ratio for each of the contributing factors was estimated separately (univariate analysis) or modelled together (multivariate analysis).

Univariate analysis identified the methylation status of *ABCB1*, *FOXC*1 and *GSTP1 *as significant predictors of overall survival. Estrogen receptor status as well as *TP53 *status and grade were also significant predictors of survival in univariate analysis (Table 1). To investigate if the methylation markers *ABCB1*, *FOXC1 *and *GSTP1 *were independent prognostic markers, we performed multivariate analysis with the methylation markers, grade, estrogen receptor status, *TP53 *status and stage. This analysis showed that the patients in this cohort with unmethylated *GSTP1 *(HR: 7.52, CI: 1.76-32.07, p = 0.006) and *FOXC1 *(HR: 7.32, CI: 1.11-48.31, p = 0.039) showed a higher risk of dying from breast cancer compared with patients methylated for the same genes (Table 2). The effect of *ABCB1 *methylation on survival was no longer significant in the multivariate analysis probably due to its association with other histopathological factors (Additional File 2). Inclusion of the operation status (HR: 2.1, p = 0.452) in the multivariate analysis did slight reduce the hazard ratio for *GSTP1 *(HR: 5.8, p = 0.028) while increasing the HR for *FOXC1 *(HR: 8.3, p = 0.03). The HR for the other parameters remained unchanged.

**Table 1:** Univariate survival analysis

		Univariate			
		
Covariate	Baseline	Coefficient	HR	95,0% CI for HR	p-value
Grade 2	Grade 1	2.047	7.742	(1.012-59.228)	0.049
Grade 3	Grade 1	2.955	19.193	(2.455-150.003)	0.005
T3	T2	-0.18	0.982	(0.128-7.517)	0.986
T4	T2	0.569	1.767	(0.228-13.717)	0.586
N1	N0	0.696	2.007	(0.696-5.783)	0.197
N2	N0	0.759	2.136	(0.714-6.383)	0.174
M	M0	0.606	1.833	(0.683-4.918)	0.229
Stage 3	Stage2	0.852	2.344	(0.686-8.008)	0.174
Stage 4	Stage2	1.088	2.970	(0.708-12.463)	0.137
ER	ER positive	1.335	3.800	(1.566-9.223)	0.003
PR	PR positive	0.620	1.859	(0.800-4.318	0.149
ErbB2	ErbB2 positive	0.965	2.624	(0.931-7.395)	0.068
*TP53*	Wild type *TP53*	1.230	3.423	(1.553-7.542)	0.002
*ABCB1*_2	*ABCB1*_2 Methylated	1.147	3.147	(1.389-7.133)	0.006
*FOXC1*_3	*FOXC1*_3 Methylated	1.030	2.802	(1.127-6.969)	0.027
*GSTP1*_2	*GSTP1*_2 Methylated	1.215	3.369	(1.280-8.868)	0.014

Univariate survival analysis using the Cox regression model using the categorical methylation data. Positive hazard ratios indicate an increased risk of dying from breast cancer and are calculated for the different covariates in reference to the baseline as given in the 2nd column.

**Table 2:** Multivariate survival analysis

		Multivariate			
		
Covariate	Baseline	Coefficient(b_i_)	HR(exp(b_i_)	95,0% CI for Exp(B)	p-value
Grade 2	Grade1	1,67	5,30	(0,52-53,75)	0,159
Grade 3	Grade1	3,45	31,65	(2,47-404,27)	0,008
ER	ER positive	2,59	13,39	(2,62-68,50)	0,002
*TP53*	TP53 wild type	1,75	5,73	(1,11-29,40)	0,036
Stage 3	Stage2	0,19	1,21	(0,20-7,03)	0,833
Stage 4	Stage2	2,00	7,38	(1,12-48,55)	0,038
*ABCB1*_2	Methylated *ABCB1*_2	-1,13	0,32	(0,05-2,20)	0,247
*FOXC1*_3	Methylated *FOXC1*_3	1,99	7,32	(1,11-48,31)	0,039
*GSTP1*_2	Methylated *GSTP1*_2	2,02	7,52	(1,76-32,07)	0,006

Multivariate survival analysis using the Cox regression model using the categorical methylation data. Positive hazard ratios indicate an increased risk of dying from breast cancer and are calculated for the different covariates in reference to the baseline as given in the 2nd column.

In order to identify the model with the minimum number of covariates that fitted best the experimental data, we used the Akaike information criterion. The best model with a reduced number of covariates explaining survival included the methylation status of *FOXC1 *and *GSTP1*, stage, grade and estrogen receptor status (Additional File 3A). The best model with a minimum number of covariates where all covariates were independent of each other included ER, grade and the *GSTP1 *methylation status (Additional File 3B). Using only a single covariate to model the survival of the patients by the AIC, the methylation status of any of the three different genes performed superior compared to the classical parameters with *GSTP1 *fitting the model best followed by *FOXC1 *and *ABCB1*. To investigate the effect of the combination of the methylation status of two genes on survival, multivariate Cox regression analysis was again performed. Only the *GSTP1/FOXC1 *pair (p = 0.005 and p = 0.013 for the combination of either one or both genes being unmethylated, respectively) remained significant together with high grade (p = 0.002) and ER status (p = 0.001) (data not shown).

## Discussion

In the presented study we analyzed the methylation patterns in the promoter regions of fourteen genes in 75 pre-treatment samples from locally advanced breast cancers, six normal tissues and six widely used cell lines. Aberrant methylation events were detected in eleven out of the fourteen genes investigated. Discussion of the negative results can be found in the Additional File 4. Due to the highly quantitative nature of the employed pyrosequencing technology and its limit of detection (~5% methylation in a sample) all methylation events detected in this study are occurring in a significant number of cells of a tumour sample and are therefore likely to have an impact on the characteristics of the tumour and - as pre-treatment samples were analyzed - might influence the response to a given chemotherapy.

The identified methylation patterns were non-random and some of the genes displayed a significant degree of co-methylation pointing to a common epigenetic mechanism for their inactivation during tumourigenesis. There was a tendency for a lower frequency of aberrant promoter methylation in basal- and normal-like tumour samples. In a study recently published, basal-like tumour cell lines were characterized by the concomitant hypermethylation of a six gene panel (*CDH1*, *CEACAM6*, *CST6*, *ESR1*, *LCN2*, *SCNN1A*) [25]. However, using methylation in repetitive elements (*LINE1/ALU*) as a surrogate for genome-wide methylation levels, basal-like breast tumours are characterized by an overall loss of methylation (J. Tost, unpublished). The observed hypomethylation in the far upstream region of *BRCA1 *(green squares for *BRCA1*_1 in Figure 1A) was found mainly in estrogen receptor negative tumours like the basal-like tumours further supporting the hypothesis that the genome-wide hypomethylation observed in breast cancer (as well as in any other cancer types) might be more pronounced in this tumour subclass. We did not detect hypermethylation of the *BRCA1 *promoter (Amplicons *BRCA1*_2 and *BRCA1*_3 in Figure 1A), which might be explained by the absence of the rare metaplastic subtype of basal-like breast cancers, to which most methylation events of *BRCA1 *seem to be restricted [26].

Since the tumour sub-classification based on gene expression is driven to a significant extent by expression of the estrogen receptor (*ESR1*), we studied its promoter methylation in normal samples as well as in a subset of the tumours. It has previously been shown that its degree of DNA methylation did not correlate well with hormone receptor status [27]. Our data confirms recently published data showing a certain degree of methylation of the estrogen receptor in tumours as well as in peritumoural/normal tissue but no difference in the quantitative distribution between normal and tumoural tissue [28].

Another studied gene,*PPP2R2B *on 5q31-q32 encodes the regulatory subunit of the protein phosphates 2A complex (PP2A) and has been proposed as a tumour suppressor gene candidate due to its negative control of cell growth and the high frequency of LOH in breast cancers [29]. An association of methylation levels to *TP53 *mutation status is reported here for the first time and might provide an alternative molecular mechanism for gene inactivation, as also the LOH has previously been associated with *TP53 *mutations [30]. The previously observed association with hormone receptor status [18] was also confirmed in our study.

### Methylation and treatment response

Our study is the first to show DNA methylation of the *ABCB1 *CpG island to be associated with *ERBB2 *amplification, *TP53 *mutation status, and response to doxorubicin treatment and overall survival in a doxorubicin-exposed cohort of primary breast cancers. Although the number of patients with progressive disease in the current study is limited and requires confirmation in other patient cohorts treated with anthracycline based treatment, there is good evidence that methylation of *ABCB1 *plays an important role in the response to doxorubicin. Lack of methylation in the central part of the *ABCB1 *CpG island was found to be associated with the *TP53 *mutation status and in particular with mutations in the L2/L3 DNA binding domain which have previously been associated with lack of response to treatment in the same patient cohort [24]. This finding further substantiates previous evidence indicating a link between *TP53 *and *ABCB1 *[31]. How much this association contributes to the resistance to doxorubicin observed in some breast cancer patients needs further investigation. Expression of *ABCB1 *has been shown to reduce intracellular doxorubicin concentration in cell cultures [32] and re-expression and promoter demethylation has been associated with resistance to anticancer drugs in vitro [33]. Although evidence from *in vivo *studies has been conflicting [34], a recent mouse model study lends support to the findings of our study by demonstrating that an increase of *ABCB*1 expression the mice leads to the development of doxorubicin resistance that might be reversed by ABCB1 inhibitors such as tariquidar [35].

### Methylation and ERBB2 overexpression

A possible link between ERBB2 and ABCB1 expression has been observed in a multidrug resistant MCF-7 cell line [36]. The amplification of the topoisomerase IIα (*TOP2A*) gene significantly improves the outcome of anthracycline based adjuvant chemotherapy [37,38]. Interestingly, *TOP2A *and *ERBB2 *are co-amplified in our dataset (p = 0.008, results not shown) warranting further investigation to explore the interaction between *ABCB1 *methylation status and *TOP2A*/*ERBB2 *amplification and how the combined effect of these proteins contributes to the drug resistance observed in anthracycline treatment regimens.

*IGF2 *exerts its action on cellular growth through the insulin-like growth factor 1 receptor which interferes with anti-ERBB2 treatment through Akt signalling [39]. In murine cancer models methylation changes in the differentially methylated region 2 of *IGF2 *have been associated with overexpression of *IGF2 *[40], which in turn might activate IGF1R signalling and increase cell growth. Here we show that hypermethylation of the DMR2 of *IGF2 *is specifically observed in ERBB2 positive breast cancers providing a new potential mechanistic link between IGF1R expression and ERBB2 status via *IGF2 *methylation status.

### Methylation and survival

We identify here the *GSTP1 *and *FOXC1 *methylation status as independent prognostic markers for breast cancer survival in a uniform patient cohort receiving neoadjuvant doxorubicin monotherapy prior to surgery and five years of tamoxifen for all ER positive patients according to a clinical study protocol [24]. *FOXC1 *methylation status might be a general prognostic marker as it is able to separate patients in good and poor survival groups in the doxorubicin treated as well as in validation cohort while *GSTP1 *and *ABCB1 *methylation status might be a predictive marker for doxorubicin monotherapy as the methylation status of these genes were not able to separate patients into survival groups in the validation cohort [2]. This is further supported by the fact that the hazard ratio for *GSTP1 *methylation decreased when the operation status was taken into account indicating that those tumours that increased further or at least did not regress during neoadjuvant treatment were more often unmethylated for *GSTP1 *while *FOXC1 *hazard ratio increased as would be expected for a treatment independent effect. *ABCB1 *methylation status proved to be a marker for survival in the discovery cohort although it was not independent of other known markers in a multivariate model. The association with survival was less significant when the expression status instead of the DNA methylation status was analysed due to a strong correlation between DNA methylation and expression for *GSTP1 *only.

We confirm here a very recent report on the presence and extent of DNA methylation in the promoter of *FOXC1*, a member of the forkhead protein family, many members of which are involved in the development and progression of cancer [41]. Mutations in *FOXC1 *have recently been reported in a candidate re-sequencing approach of breast tumours [42] and *FOXC1 *was found to be specifically hypomethylated and highly expressed in CD44+ compared to CD24+ stem cell progenitors, but no data correlating survival to the methylation patterns was presented [43]. The overexpression of the closely related *FOXC2 *gene has been found to promote tumour metastasis and invasiveness [44].

CpG hypermethylation of the promoter region of the *glutathione-S-transferase 1 *(*GSTP1*) is a well established biomarker for hormone dependent cancers. Low activity of *GSTP1 *resulting from promoter hypermethylation may increase the effective therapeutic dose of the pharmacological agent due to lower conjugation and inactivation of the drug leading to increased survival. In support of this hypothesis it has been shown that *GSTP1 *expression correlates with doxorubicin resistance in breast cancer cell lines [45]. The observed improved survival has very recently been shown in samples in concordance with previous reports where the absence of GSTP1 protein expression correlated with improved survival in invasive breast cancer samples [46,47].

## Conclusions

Methylation at the *ABCB1 *or *GSTP1 *promoter improved overall survival probably due to prolonged availability and activity of the drug in the cell while *FOXC1 *methylation might be a protective factor against tumour invasiveness.

The *FOXC1 *methylation status might be a widely applicable prognostic factor for breast cancer patients while the methylation status of *ABCB1 *and *GSTP1 *might be a predictive factor for doxorubicin and perhaps anthracycline treatment in general. However, further studies are necessary to confirm the predictive value of these markers requiring additional patient cohorts treated with a doxorubicin/anthracycline based monotherapy. Valuable time for treatment might be gained and the serious side-effects of a doxorubicin based regimen might be avoided taking the methylation status for treatment decisions into account. As the analyzed cohort consists of locally advanced primary tumours, it will be interesting to investigate the DNA methylation profiles also in T1/T2 and early stage breast cancer samples. Despite similar RNA expression profiles [21], some biological differences such as different frequencies of polymorphic alleles have recently been found to be enriched in advanced tumours [48]. Additional studies including prospective trials are required to fully evaluate the potential of these promising DNA methylation based markers to predict and monitor the efficacy of chemotherapy and to measure their impact on breast cancer management.

## Methods

### Patient cohorts

#### Discovery cohort (Doxorubicin treated)

Locally advanced breast cancer patients, admitted to the Haukeland University Hospital in Bergen (Norway) between 1991 and 1998 were part of a prospective study evaluating predictive factors for response to doxorubicin (n = 94). Tumour DNA was available in sufficient quantity to perform methylation analyses from 75 of the patients. Tissue was obtained by an incisional biopsy prior to therapy and was immediately snap-frozen (liquid nitrogen in the theater) as previously reported [24]. DNA was isolated from snap frozen tumour tissue using phenol/chloroform extraction. The primary treatment consisted of weekly doxorubicin treatment (14 mg/m^2^) scheduled for 16 weeks. Patients with an operable tumour (n = 60) after neoadjuvant treatment had surgery followed by radiotherapy immediately after termination of the neoadjuvant chemotherapy. Eight patients had to be given radiotherapy prior to surgery due to local tumour extension, and seven patients were not eligible for surgery and were treated on an individual basis. Women with estrogen and/or progesterone positive tumours were all treated with tamoxifen (30 mg daily for 5 years). The main clinical characteristics of the analyzed 75 samples are given in Table 3. The study protocol was approved by the local ethical committee, and the patients gave their informed consent.

**Table 3:** Molecular and clinical characteristics of the analyzed sample cohort

Clinicopathological factors	Number of samples
**Median age at diagnosis**	65 (range 32-85)
	
**Histological grade**	
Grade 1	18 (24%)
Grade 2	38 (50.7%)
Grade 3	19 (25.3%)
	
**Response**	
Progressive Disease	7 (9.5%)
PR, MC or SD	67 (90.5%)
	
**Tumor size**	
T2	3 (4%)
T3	47 (62.7%)
T4	25 (33.3%)
	
**Lymph node metastasis**	
N0	25 (33.3%)
N1	29 (38.7%)
N2	21 (28%)
	
**Distant metastasis**	
M0	66 (88.0%)
M1	9 (12%)
	
**Stage**	
Stage 2	18 (24%)
Stage 3	46 (61%)
Stage 4	11 (15%)
	
***TP53 *mutations**	
Wild type	55 (73.3%)
Mutant	20 (26.7%)
	
**Estrogen receptor status**	
Positive	65 (86.7%)
Negative	10 (13.3%)
	
**Progesteron receptor status**	
Positive	58 (77.3%)
Negative	17 (22.7%)
	
***ErbB2 *receptor status**	
Positive	11 (25%)
Negative	33 (75%)
	
**Survival**	
> 5 years	20 (26.7%)
< 5 years	55 (73.3%)

PR: partial response; MC: minimal change; SD: stable disease. Lymph node status was assessed clinically prior to neoadjuvant therapy and does not necessarily correspond to pathological lymph node status. In this context N0 means that no enlarged nodes were felt prior to therapy. N1 corresponds to the presence of palpable and movable ipsilateral axillary lymph nodes suspicious of the presence of metastases while N2 corresponds to fixed ipsilateral axillary lymph nodes and thus very likely to the presence of tumours.

#### Validation cohort

163 random, unselected breast cancer samples were used for the validation of the observed associations with clinicopathological factors. Clinical and molecular parameters such as histological grade (n = 162), Estrogen receptor status (n = 158) and *TP53 *mutation status (n = 162) were available and used for validation. Follow-up/survival data was available for 87 of the patients. Tumour DNA extraction, bisulphite treatment and pyrosequencing analyses was performed using the same procedures as for the discovery dataset.

#### Normal material

DNA samples from normal breast tissue were included as control samples for methylation analysis. Normal breast tissue (n = 6) was obtained from women who underwent a biopsy of the mammary gland because of mammographic abnormalities, but for which histology confirmed the presence of only normal tissue.

#### Cell lines

The sample set was completed by immortalized human mammary epithelial cells (HMEC) and five widely used breast cancer cell lines (BT474, MCF-7, MDA-MB-231, SK-BR-3, and T47D).

### *TP53* mutation, copy number and expression analyses

Mutations in *TP53 *were analyzed in both the discovery and the validation cohorts by temporal temperature gradient electrophoresis (TTGE) followed by Sanger sequencing as previously described with primers covering regions (exons and introns) from exon 2-11 [24,49]. 50 of the doxorubicin treated tumours have been analyzed for gene expression using genome wide cDNA microarrays [21], and a subset of these tumours was analyzed for copy number alterations [50].

### Methylation assays

Assays were optimized on unmethylated and methylated DNA as previously described [51]. DNA concentrations were determined using the Quant-iT™ dsDNA broad range assay kit (Invitrogen, Cergy Pontoise, France) and normalized to a concentration of 50 ng/μl. One μg of DNA was bisulphite converted using the MethylEasy™ HT Kit for Centrifuge (Human Genetic Signatures, North Ryde, Australia) according to the manufacturer's instructions. Quantitative DNA methylation analysis of the bisulphite treated DNA was performed by pyrosequencing or - in case of several sequencing primers - by serial pyrosequencing [51]. Regions of interest were amplified using 30 ng of bisulfite treated human genomic DNA and 5 to 7.5 pmol of forward and reverse primer, one of them being biotinylated. Oligonucleotides for PCR amplification and pyrosequencing (Additional File 5) were synthesized by Biotez (Buch, Germany). Reaction conditions were 1× HotStar Taq buffer supplemented with 1.6 mM MgCl_2_, 100 μM dNTPs and 2.0 U HotStar Taq polymerase (Qiagen, Courtaboeuf, France) in a 25 μl volume. The PCR program consisted of a denaturing step of 15 min at 95°C followed by 50 cycles of 30 s at 95°C, 30 s at the respective annealing temperature (Additional File 1) and 20 s at 72°C, with a final extension of 5 min at 72°C. 10 μl of PCR product were rendered single-stranded as previously described [51] and 4 pmol of the respective sequencing primer (Additional File 1) were used for analysis. Quantitative DNA methylation analysis was carried out on a PSQ 96MD system with the PyroGold SQA Reagent Kit (Pyrosequencing) and results were analyzed using the Q-CpG software (V.1.0.9, Pyrosequencing AB).

### Expression analysis

50 of the tumours have previously been analyzed for gene expression using genome wide cDNA microarrays [21]. For quantitative RT-PCR based expression analysis (TaqMan), cDNA was synthesized from 1 μg of total RNA with random hexamers using the High Capacity cDNA Reverse Transcription Kit (Applied Biosystems, Foster City, Ca) in a final volume of 10 μl. Real-time PCR reactions were performed in triplicate in a final volume of 10 μl using 50 ng of cDNA and the TaqMan^® ^Gene Expression Master Mix (Applied Biosystems). TaqMan assays were all purchased from Applied Biosystems: Hs 00943351_g1 (*GSTP1*), Hs00184500_m1 (*ABCB1*) and Hs00559473_s1 (*FOXC1*). Human Breast Total RNA (Ambion, Austin, TX) was used to generate standard curves. *PMM1 *(Hs00963626_m1) was used as endogenous control and the relative gene expression levels were determined using the standard curve method and normalized to *PMM1*.

### Statistical analysis

Differences in the presence of methylation were determined by a two-sided Fisher's test and χ^2 ^tests. Samples were scored as methylated when the methylation degree exceeded the average methylation degree of the normal samples by two times the standard deviation of the normal samples and had at least a methylation degree of 5% (detection limit of the technology). Odds ratio and 95% confidence intervals were calculated. Differences in the distribution of methylation were assessed by the non-parametric Mann-Whitney or the Kruskal-Wallis test. Correlation between the methylation status of the different genes was calculated by the non-parametric Kendall's tau test. Pearson's coefficients were used to study the correlation between methylation and expression levels. All calculations were performed using Statistical Package for Science version 15.0. The Cox proportional hazards model was used to evaluate the effect sizes (given as hazard ratios), 95% Confidence intervals (CI), regression coefficients and statistical significance of known clinicopathological features as well as the methylation status of selected genes. All covariates were treated as categorical variables. To investigate the relationship between multiple explanatory factors and survival, we used the Akaike information criterion (AIC) [52]. AIC evaluates the suitability of a selection of covariates in order to model the experimental observation and adds a penalty score with increasing number of parameters included in the model. The model with the minimum AIC is thus the model describing best the survival data. All possible combinations with respect to grade, stage, ER and *TP53 *mutation status as well as methylation of *ABCB1*, *FOXC1 *and *GSTP1 *respectively, were considered as covariates to the model. With *L *being the likelihood function of the model and *k *indicating the number of parameters of the model, the Akaike information criterion (AIC) is calculated by: AIC = -2log *L*+2*k*.

## Competing interests

The authors declare that they have no competing interests.

## Authors' contributions

ED and JAR performed laboratory experiments and data analyses and participated in writing of the manuscript. HS was involved in the statistical analyses. PEL, SG and TA were responsible for the doxorubicin treated patient cohort and clinical database management. ALBD and VNK were responsible for the validation cohorts and IB for the control samples. IGG, ALBD, VNK and JT initiated and designed the study. JT wrote the manuscript and VNK, PEL and ALBD participated in writing the manuscript. All authors have read and approved the final manuscript.

## Supplementary Material

Additional file 1 **Correlation between DNA methylation and RNA expression for *GSTP1*, *FOXC1 *and *ABCB1***. Scatter plots showing the correlation between DNA methylation and RNA expression as measured by TaqMan for *GSTP1*, *FOXC1 *and *ABCB1*. Click here for file

Additional file 2 **Associations of the presence/absence and degree of methylation and the clinical and molecular parameters**. Associations of the presence/absence and degree of methylation and the clinical and molecular parameters of the samples by Fisher's exact test (2 categorical variables) or χ^2 ^analysis (3 categorical variables) with odds ratio (OR) and 95% Confidence interval (CI) and their respective p-value in the validation cohort. Statistical significance of the differences in the distribution of the degree of methylation is assessed by the non-parametric Mann-Whitney and Kruskal-Wallis test. Samples are called methylated if the methylation degree exceeded 5% and the average methylation degree of the healthy tissue samples plus at least two times the standard deviation of the healthy tissues. Click here for file

Additional file 3 **Best models to fit the observed survival data**. A) Best models with a varying number of covariates to fit the observed survival data. B) Multivariate analysis of the best model with the minimum number of covariates. The presented model is the only one where all covariates are significant in multivariate analysis. Click here for file

Additional file 4 **Supplementary discussion**. Discussion of negative results. Click here for file

Additional file 5 **PCR and pyrosequencing primers**. Sequences of primers used for amplification and pyrosequencing reactions, Genbank accession numbers and nucleotides (Nt) corresponding to the amplified fragments as well as the annealing temperatures for the respective PCR amplifications. CpGs are numbered in the order of appearance from the 5' end of an amplification product. Y = pyrimidine. Click here for file
