# Supplementary figures and images for: DNA methylation profiling in doxorubicin treated primary locally advanced breast tumours identifies novel genes associated with survival and treatment response
Source: Mol Cancer. 2010 Mar 25;9:68. doi: 10.1186/1476-4598-9-68 (PMC2861056; doi:10.1186/1476-4598-9-68)

*GSTP1*

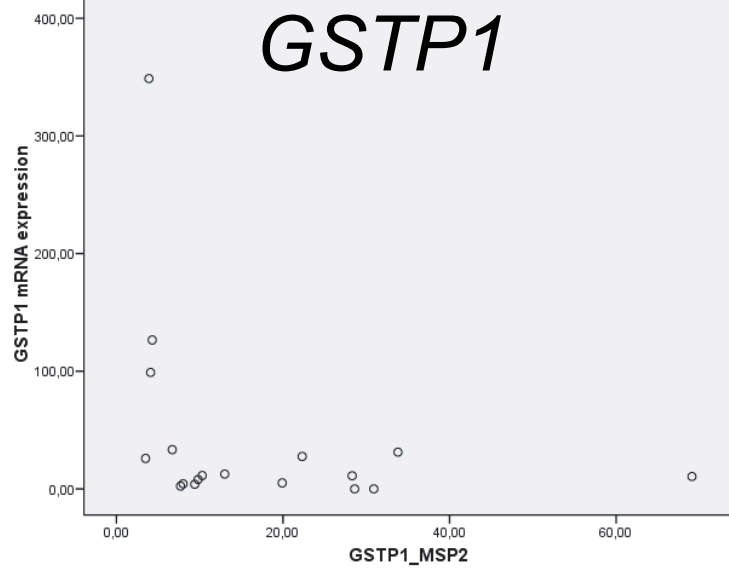

*FOXC1*

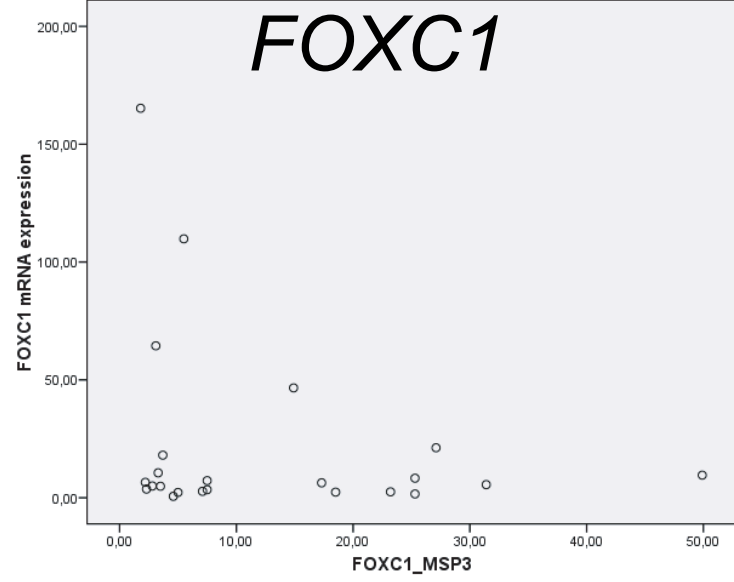

*ABCB1*

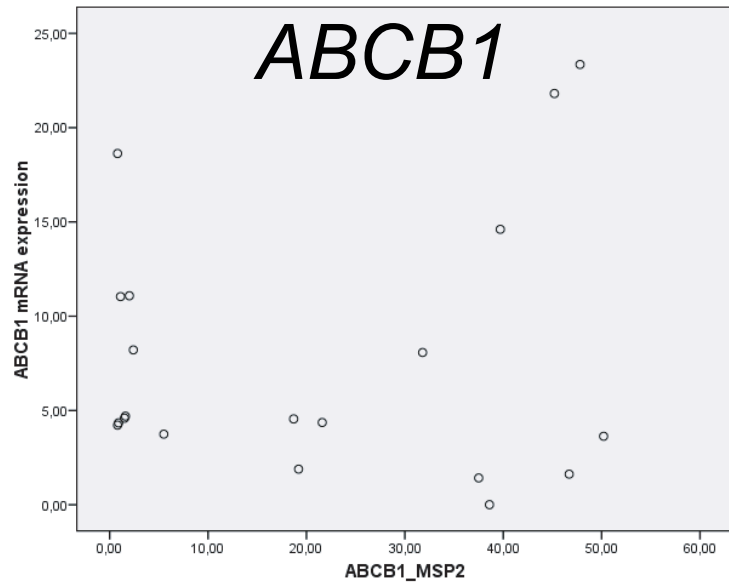

Supplement: Additional file 1 — Correlation between DNA methylation and RNA expression for GSTP1, FOXC1 and ABCB1. Scatter plots showing the correlation between DNA methylation and RNA expression as measured by TaqMan for GSTP1, FOXC1 and ABCB1. [file 1476-4598-9-68-S1.PDF]
