# Supplementary material for: DNA methylation profiling in doxorubicin treated primary locally advanced breast tumours identifies novel genes associated with survival and treatment response
Source: Mol Cancer. 2010 Mar 25;9:68. doi: 10.1186/1476-4598-9-68 (PMC2861056; doi:10.1186/1476-4598-9-68)
Supplement: Additional file 2 — Associations of the presence/absence and degree of methylation and the clinical and molecular parameters. Associations of the presence/absence and degree of methylation and the clinical and molecular parameters of the samples by Fisher's exact test (2 categorical variables) or χ2 analysis (3 categorical variables) with odds ratio (OR) and 95% Confidence interval (CI) and their respective p-value in the validation cohort. Statistical significance of the differences in the distribution of the degree of methylation is assessed by the non-parametric Mann-Whitney and Kruskal-Wallis test. Samples are called methylated if the methylation degree exceeded 5% and the average methylation degree of the healthy tissue samples plus at least two times the standard deviation of the healthy tissues. [file 1476-4598-9-68-S2.PDF]

Additional file 2 - Associations of the presence/absence and degree of methylation and the clinical and molecular parameters

		Discovery cohort					Validation cohort				
					p-value					p-value	
Gene	Clinicopathological factor	Methylation Status		Odds ratio (95% CI)	Fisher	Non-parametric	Methylation Status		Odds ratio (95% CI)	Fisher	Non-parametric
	**Histological grade**										
PPP2R2B_3		Unmethylated	Methylated				Unmethylated	Methylated			
	Grade 1	8	9				1	3			
	Grade 2	6	26	NA	0.019	0.02	25	68	NA	0.008	0.013
	Grade 3	9	7				25	22			
	**Treatment response**										
*ABCB1*_2		Unmethylated	Methylated				Unmethylated	Methylated			
	Progressive disease	6	1	13.50 (1.52-119.6)	0.0076	0.04	NA	NA	NA	NA	NA
	PR,MD or SD	20	45				NA	NA			
	***TP53* mutations**										
*ABCB1*_2		Unmethylated	Methylated				Unmethylated	Methylated			
	Mutant	11	8	3.58 (1.17-10.35)	0.028	0.031	25	17	2.49 (1.21-5.14)	0.018	0.012
	Wild type	15	38				43	73			
PPP2R2B_3		Unmethylated	Methylated				Unmethylated	Methylated			
	Mutant	11	7	4.58 (1.44-14.5)	0.010	0.028	23	16	3.81 (1.79-8.22)	0.001	0.006
	Wild type	12	35				29	77			
	**Estrogen receptor**										
PPP2R2B		Unmethylat	Methy				Unmethylated	Methylated			

_3		ed	lated								
	Negative	9	1	15.65 (1.88-131.5)	0.004	0.02	21	17	3.01 (1.39-6.48)	0.006	0.004
	Positive	23	40				30	73			
	**ErbB2**										
ABCB1_2		Unmethylated	Methylated				Unmethylated	Methylated			
	Positive	9	2	10.50 (1.88-58.6)	0.005	0.0019	NA	NA	NA	NA	NA
	Negative	9	21				NA	NA			
IGF2_DMR2		Hypo Hyper	Meth				Unmethylated	Methylated			
	Negative	20 4	8	NA	0.007	0.036	NA	NA	NA	NA	NA
	Positive	2 6	2				NA	NA			

Associations of the presence/absence and degree of methylation and the clinical and molecular parameters of the samples by Fisher's exact test (2 categorical variables) or χ^2 analysis (3 categorical variables) with odds ratio (OR) and 95 % Confidence interval (CI) and their respective p-value in the validation cohort. Statistical significance of the differences in the distribution of the degree of methylation is assessed by the non-parametric Mann-Whitney and Kruskal-Wallis test. Samples are called methylated if the methylation degree exceeded 5 % and the average methylation degree of the healthy tissue samples plus at least two times the standard deviation of the healthy tissues.
