# Supplementary material for: DNA methylation profiling in doxorubicin treated primary locally advanced breast tumours identifies novel genes associated with survival and treatment response
Source: Mol Cancer. 2010 Mar 25;9:68. doi: 10.1186/1476-4598-9-68 (PMC2861056; doi:10.1186/1476-4598-9-68)
Supplement: Additional file 3 — Best models to fit the observed survival data. A) Best models with a varying number of covariates to fit the observed survival data. B) Multivariate analysis of the best model with the minimum number of covariates. The presented model is the only one where all covariates are significant in multivariate analysis. [file 1476-4598-9-68-S3.PDF]

Additional File 3

A) Best models with a varying number of covariates to fit the observed survival data. B) Multivariate analysis of the best model with the minimum number of covariates. The presented model is the only one where all covariates are significant in multivariate analysis.

A

**# covariates**	**Covariate 1**	**Covariate 2**	**Covariate 3**	**Covariate 4**	**Covariate 5**	**Covariate 6**	**Covariate 7**	**-2loglikelihood**	**AIC**
7	Grade	ER	*TP53*	*ABCB1_2*	*FOXC1_3*	*GSTP1_2*	Stage	84.6349	100.6349
6	Grade	ER	*TP53*	*FOXC1_3*	*GSTP1_2*	Stage		93.4613	107.4613
5	Grade	ER	*FOXC1_3*	*GSTP1_2*	Stage			94.5062	106.5062
4	Grade	ER	*GSTP1*	Stage				110.4242	120.4242
3	Grade	ER	*GSTP1*					112.8838	120.8838

B

	**Multivariate**			
**Parameter**	**Coefficient(b_i)**	**HR(exp(b_i)**	**95,0% CI for Exp(B)**	**p-value**
ER	2.540893249	12.69100213	(3.51-45.91)	0.00010738
GSTP1_MSP2	1.666497664	5.293595346	(1.81-15.48)	0.00233268
Grade (vs Grade 1)	3.137068713	23.03624196	(2.40-220.43)	0.00648169
Grade (vs Grade 2)	4.644355632	103.9963323	(8.76-1235.20)	0.00023468
