# Supplementary material for: DNA methylation profiling in doxorubicin treated primary locally advanced breast tumours identifies novel genes associated with survival and treatment response
Source: Mol Cancer. 2010 Mar 25;9:68. doi: 10.1186/1476-4598-9-68 (PMC2861056; doi:10.1186/1476-4598-9-68)
Supplement: Additional file 4 — Supplementary discussion. Discussion of negative results. [file 1476-4598-9-68-S4.PDF]

Supplementary discussion:

Important negative results:

A multitude of associations of gene-specific DNA methylation and the different molecular and clinical parameters of the analyzed samples were found, some of them confirming previous studies while other ones are reported here for the first time. We did not find any methylation in the *ATM* promoter region which is in concordance with another recent report showing that previous positive findings for the presence of methylation were caused by a suboptimal design of the primers used for methylation-specific PCR [1, 2]. No DNA methylation was also detected in the promoter region of the chemokine receptor *CXCR4,* an important regulator of breast cancer metastasis [3]. So far the presence of DNA methylation had not been described for primary breast tumors or breast cancer cell lines. We did not observe any methylation in the promoter region of *FBXW7*, a *TP53* dependent tumor suppressor gene acting as ubiquitin ligase involved in the degradation of phosphorylated cyclin E and mTOR [4]. Extensive genetic screens have established its role as a tumor suppressor gene and somatic alterations have been found in various cancers including breast cancer [5, 6]. No inactivation by epigenetic mechanisms has so far been reported and our data supports the hypothesis of solely genetic mechanisms for its inactivation. *CDH3* (*P-cadherin*) is a marker of basal-like breast carcinomas [7] and its overexpression is at least partially caused by loss of DNA methylation in the promoter CpG island, which has been proposed as a marker for poor prognosis in invasive breast carcinomas [8]. Analyzing three different regions of the promoter CpG island including the previously reported one we were not able to detect methylation, neither in the cell lines nor in the normal breast tissues or the primary tumors which questions both the methylation of its promoter in normal tissue and its demethylation during carcinogenesis. Loss of the negative regulator of the Akt pathway *PTEN* is either caused by mutations but occurs also frequently through promoter hypermethylation

paticularly in estrogen positive breast tumors [9]. These results were confirmed in our study, however, neither of the previously reported associations of *PTEN* methylation with the absence [9] or the presence [10] of *ERBB2* amplification was confirmed in the present study possibly due to the low number of ERBB2 positive tumors.

1. Brandes JC, Carraway H, Herman JG: **Optimal primer design using the novel primer design program: MSPprimer provides accurate methylation analysis of the ATM promoter.** *Oncogene* 2007, **26:**6229-6237.
2. Vo QN, Kim WJ, Cvitanovic L, Boudreau DA, Ginzinger DG, Brown KD: **The ATM gene is a target for epigenetic silencing in locally advanced breast cancer.** *Oncogene* 2004, **23:**9432-9437.
3. Smith MC, Luker KE, Garbow JR, Prior JL, Jackson E, Piwnica-Worms D, Luker GD: **CXCR4 regulates growth of both primary and metastatic breast cancer.** *Cancer Res* 2004, **64:**8604-8612.
4. Mao JH, Kim IJ, Wu D, Climent J, Kang HC, DelRosario R, Balmain A: **FBXW7 targets mTOR for degradation and cooperates with PTEN in tumor suppression.** *Science* 2008, **321:**1499-1502.
5. Chanock SJ, Burdett L, Yeager M, Llaca V, Langerod A, Presswalla S, Kaaresen R, Strausberg RL, Gerhard DS, Kristensen V, et al: **Somatic sequence alterations in twenty-one genes selected by expression profile analysis of breast carcinomas.** *Breast Cancer Res* 2007, **9:**R5.
6. Akhoondi S, Sun D, von der Lehr N, Apostolidou S, Klotz K, Maljukova A, Cepeda D, Fiegl H, Dafou D, Marth C, et al: **FBXW7/hCDC4 is a general tumor suppressor in human cancer.** *Cancer Res* 2007, **67:**9006-9012.
7. Fadare O, Tavassoli FA: **Clinical and pathologic aspects of basal-like breast cancers.** *Nat Clin Pract Oncol* 2008, **5:**149-159.
8. Paredes J, Albergaria A, Oliveira JT, Jeronimo C, Milanezi F, Schmitt FC: **P-cadherin overexpression is an indicator of clinical outcome in invasive breast carcinomas and is associated with CDH3 promoter hypomethylation.** *Clin Cancer Res* 2005, **11:**5869-5877.
9. Perez-Tenorio G, Alkhori L, Olsson B, Waltersson MA, Nordenskjold B, Rutqvist LE, Skoog L, Stal O: **PIK3CA mutations and PTEN loss correlate with similar prognostic factors and are not mutually exclusive in breast cancer.** *Clin Cancer Res* 2007, **13:**3577-3584.
10. Garcia JM, Silva J, Pena C, Garcia V, Rodriguez R, Cruz MA, Cantos B, Provencio M, Espana P, Bonilla F: **Promoter methylation of the PTEN gene is a common molecular change in breast cancer.** *Genes Chromosomes Cancer* 2004, **41:**117-124.
