# Supplementary material for: DNA methylation profiling in doxorubicin treated primary locally advanced breast tumours identifies novel genes associated with survival and treatment response
Source: Mol Cancer. 2010 Mar 25;9:68. doi: 10.1186/1476-4598-9-68 (PMC2861056; doi:10.1186/1476-4598-9-68)
Supplement: Additional file 5 — PCR and pyrosequencing primers. Sequences of primers used for amplification and pyrosequencing reactions, Genbank accession numbers and nucleotides (Nt) corresponding to the amplified fragments as well as the annealing temperatures for the respective PCR amplifications. CpGs are numbered in the order of appearance from the 5' end of an amplification product. Y = pyrimidine. [file 1476-4598-9-68-S5.PDF]

Additional File 5

GENE[a]	PCR primer forward	PCR primer reverse	Tem. PCR (°C)	Pyrosequencing primer	CpG number
ABCB1_1 [GenBank:AY910577] Nt 114132-114333 -859 to --657	5’- TTTTGGAAATTTAATTTGTTT	5’-Biotin-CTACTTCTTTAAACTTAAAAAAACC	53	5'- GATTGGTTGGGTAGGA	7
ABCB1_2 [GenBank:AY910577] Nt 114583-114800 -408 to -191	5’- TTAGATTTAGGAGTTTTTGGAGTAG	5’-Biotin-AAAACAAAATTAAAAATCTAACAAC	58	5'- TGGTATTGGATTATGTTGTT	10
				5'- TGGGTGGGAGGAAGT	10
ABCB1_3 [GenBank:AY910577] Nt 114826-115077 -165 to +186	5’- ATAGGAAAGTTTTTGTAGTGTTTAT	5’-Biotin-CAAACTAATTACCTTTTATTATTCAATTTA	58	5'- GGTTAGAGTAGTTGGGGTA	5
				5'- AGAGGTTAGTTTTATTTTTA	5
				5'- TAAAAATAAAACAAAA	3
ATM_1 [GenBank:AY220758] Nt 1301-1610 -549 to -283	5’- GAGGGTAATAATATGATTAAAATT	5’-Biotin-AAACTCTCACCCACCCTCTTC	56	5'- CGTTTTYGTTAGAGAAAGA	8

ATM_2 [GenBank:AY220758] Nt 1591-1808 -302 to -89	5'- AAGAGGGTGGGTGAGAGTTT	5'-Biotin-AACRCCCACTTCTAAAAAAAA	56	5'- GGTGAGAGTTTYGGAGT	12
				5'- TTGGGGTAGTGTTTTGA	8
				5'- GTTGGTTATTGGTGGATA	11
ATM_3 [GenBank:AY220758] Nt 1784 – 1953 -107 to +60	5'- GTTTTTTTTTTTAGAAGTGGG	5'-Biotin-AAAAATCAAAATAATATCAACC	56	5'- GGGYGTTGGGTAGT	8
				5'- GTTAAYGGAGAAAAGAAGT	9
BRCA1_1 [GenBank:AC135721] Nt 32624-32929 -1508 to -1202	5'- GGTTAGTTTAGAGAGGGGTTTTTATAG	5'-Biotin-TCACACTAAAAAAATTTAAAAAAATTCAAA	59	5'- GGGGTTTTTATAGYGTAGTG	10
				5' - GTATTTTGAATTATAGATTT	6
				5'- ATTAAAAYGATTAGTTGTT	3
BRCA1_2 [GenBank:AC135721] Nt 33805-34101 -324 to -30	5'- TTTTATGGTAAATTTAGGTAGAATTTT	5'-Biotin-TATCTAAAAAACCCCACAACCTATC	58	5'- TTTGAGAGGTTGTTGTTTAG	11
				5'- GGGTGGTTAATTTAGAGTTT	4
				5'- GGAATTATAGATAAATTAAA	6
BRCA1_3 [GenBank:AC135721] Nt 34447-34726 +302 to +593	5'- AGGAGTTTTAGATTAGTTTGATTAA	5'-Biotin-CTAACACCTCTTCTTCCACAAAATC	58	5'- GAGTTTTAGATTAGTTTGAT	5
				5'- GGAGGTTGAGGTAGGAGA	8

CDH3_1<br>[GenBank:AC126773]<br>Nt 138584-138801<br>+31 to +248	5’-<br>GGGTTTGTTTTAAAGTTTT<br>G	5’-Biotin-<br>TTTACAAAAACTCAACCT<br>CTATCTC	62	5'- TTTAAAGTTTTGGGATAATA	8
CDH3_2<br>[GenBank:AC126773]<br>Nt 138802-139071<br>+249 to +548	5’-<br>GAATAGTTTTAGAAAAGT<br>TAGGAGAG	5’-Biotin-<br>AAAAACCCCATAACTAC<br>AAAAAAAA	59	5'- GAGYGTAGGAGGGTATT	8
				5'- GGYGTTGGATTAATTAGTA	10
CDH3_3<br>[GenBank:AC126773]<br>+875 to +1156	5’-Biotin-<br>AAAGGTAAGATTTTTAGG<br>GTGGAAT	5’-<br>AACTAAATAAAAAACTC<br>AACCATCC	63	5'- AATCAAAATAAACAATAACT	9
CDKN2A<br>[GenBank:AF527803]<br>Nt 19893-20154<br>+118to +380	5’-<br>GAGGGGTTGGTTGGTTAT<br>TAGA	5’-biotin-<br>TACAAACCCTCTACCCAC<br>CTAAAT	64	5'- TGGTTATTAGAGGGTG	7
				5'- GAGGGGGAGAGTAGGTAG	6
				5'- GGGGAGTAGTATGGAGTTTT	8
				5'- GGGTYGGGTAGAGGA	9
CXCR4<br>[GenBank:AF005058]<br>Nt 1854-1607<br>+752 to + 1000	5’-Biotin-<br>GGTGTTAAAGGGGAGGTT<br>AAATTAT	5’-<br>ATACCAATCTCCCAACAC<br>AATAAAC	61	5'- AAACRAATAAACCCAC	7
				5'- ATAACAACAACTAAAAATTA	12
				5'- CRCRAATAAATCCTACAAT	3
ESR1_2<br>[GenBank:AY425004]<br>Nt 2426-2736	5’-<br>GTAGTTTAAGATTTTTTTG<br>GAG	5’-Biotin-<br>CCAAATAATAAAACACC<br>TACTAACC	61	5'- TATTTGGATAGTAGTAAGTT	13

+449 to +759				5'- YGTTAAYGYGTAGGTT	8
				5'- TTYGTTGATGTTATTGTATT	5
ESR1_3 [GenBank:AY425004] Nt 2858-3201 +881 to +1224	5’-Biotin-GGAGAAGGGAGAGTTTAGGGAGTTG	5’-ACCCCCAACTTTAAATACCAAAAAC	66	5'- CCCCCRAACTAAACC	13
				5'- AAAATACRTTTTTTTCAACT	11
FBXW7_1 [GenBank:AC023424] Nt 114261-113965 -1464 to -1168	5’-Biotin-TTTTAAAGGAGTAGTTTTTATTTGTTT	5’-CTAACCTTTTTCCCCCTTCCAAC	57	5'- TTTGTTTYGAAGGAAAT	7
FBXW7_2 [GenBank:AC023424] Nt 112933-112706 -948 to -649	5’-TTGAAAAGATTTAGGAAGAGGAAAAG	5’-Biotin-AAACCACTCACACTTTTAAAAAAAA	62	5'- AGGAAAAGAGGAGGT	10
				5'- GGTTGTYGTTTGGTTTA	9
				5'- GAAGGGAATTAATTTATTTT	5
FOXC1_1 [EMBL:AL034344] Nt 64529-64798 -1152 to -8568	5’-Biotin-GGTTTTTATTTTTTATTGGTTGT	5’-CCTTCCAAAAAACTCTACCCTAAAC	62	5'- GCRTCACCTTTTCTC	5
FOXC1_3 [EMBL:AL034344] Nt 65513-65727 +354 to+568	5’-TAAGTAGGGTTGGTAGAATAGTATT	5’-Biotin-ACAACCTATCCTTCTCCTCCTTATC	62	5'- TTCGGATTTTTATAATATGT	9
GSTP1_1 [GenBank:AY324387] Nt 1670-1970 +3 to +304	5’-GAAAGAGGGAAAGGTTTTTT	5’-biotin-CCATACTAAAAACTCTAAACCCCATC	58	5'- GTAGTTTTYGTTATTAGTGA	6
				5'- GGATTATTTTTATAAGGT	8

GSTP1_2 [GenBank:AY324387] Nt 1670-1970 +3 to +304	5’-biotin-GAAAGAGGGAAAGGTTTTTT	5’-CCATACTAAAAACTCTAAACCCCATC	58	5'- CGAACCTTATAAAAATAATC	7
IGF2 DMR2 [GenBank:AF517226] Nt 7978-7724- +15856 to +16111	5’-GGGAAAGGGGTTTAGGATTTTTAT	5’-biotin-ATAATTTACTCCCCCTTCAACCTC	60	5’-GATTTTTATYGGAAGTA	5
				5’-GGGTYGATAYGTTTTTTT	8
				5’-TTTTATAGTATAGAGAGAGT	3
MLH1 [GenBank:AY217549] Nt 1504-1800 -564 to -268	5’-GGGAGGTTATAAGAGTAGGGTTAA	5’-Biotin-TCTCAACTCTATAAATTACTAAATCTCTTC	61,4	5'- GAGAGGAGGAGTTTGAGA	5
				5'- TTTTTATTGGTTGGATATTT	11
				5'- AAAACGAATTAATAGGAAGA	8
PPP2R2B_1 [GenBank:AF152102] Nt 1804-2020 -445 to -229	5’-AGGTAGGGAGTTAGGGGTTTTG	5’-Biotin-CTCTTTCCCAACAACAATAATAACC	62	5'- GGGGTTTTGGGTGTT	7
				5'- ATTTGAATTTGTAAGTTAGT	7
PPP2R2B_3 [GenBank:AF152102] Nt 3012-3336 +516 to +838	5’-AGTTTGTTGTTAATGGAGGAGGATAT	5’-Biotin-AATCCCTAAAAACCATTTTAACTCC	62	5'- AATGGAGGAGGATATTGA	6
				5’-AAGGTAAYGTTAGTTT	11
				5'- GGTTTTAGGGTTG	10
				5'- CGGTAGTTTTAGTGTATT	10
PTEN_2 [GenBank:AF67844] Nt 22468-22791	5’-ATATTGGGTATGTTTAGTAGAGTTTG	5’-Biotin-CAAACTTCCATCATAACTACAACTTC	62	5'- TGGYGGGATTTTTTA	10

-98 to +227			62	5'- TGGYGGGATTTTTTA	10
				5’-TTTGGGGATTTTG	3
				5’-TTYGTATTTAGAGTTAT	12
PTEN_3 [GenBank:AF67844] Nt 22893-23149 +586 to +843	5’-TTGTTATTATTTTTAGGGTTGGGAA	5’-Biotin-CTAAACCTACTTCTCCTCAACAACC	62	5'- GTTGGTATATTTAGGGATT	13
				5'- CGTYGGYGGAGGTA	6

[a] Positions for the transcription start site are given with reference to one isoform only and should therefore be taken with care.
